# ﻿A new species of the *Cyrtodactylusquadrivirgatus* complex (Chordata, Reptilia, Squamata, Gekkonidae) from Sumatra Barat, Indonesia

**DOI:** 10.3897/zookeys.1168.98724

**Published:** 2023-07-04

**Authors:** Yuni Ahda, Fitra Arya Dwi Nugraha, Djong Hon Tjong, Nia Kurniawan, Yunico Amardi, Muhammad Alif Fauzi, Si-Min Lin

**Affiliations:** 1 Department of Biology, Faculty of Mathematics and Natural Sciences, Universitas Negeri Padang. Jl. Hamka, Kota Padang 25132, Sumatra Barat, Indonesia Universitas Negeri Padang Padang Indonesia; 2 Department of Biology, Faculty of Mathematics and Natural Sciences, Universitas Andalas, Limau Manis, Pauh, Kota Padang 25175, Sumatra Barat, Indonesia Universitas Andalas Padang Indonesia; 3 Department of Biology, Faculty of Mathematics and Natural Sciences, Universitas Brawijaya, Jl. Veteran 65145, Ketawanggede, Lowokwaru, Kota Malang, Jawa Timur, Indonesia Universitas Brawijaya Malang Indonesia; 4 Department of Life Science, National Taiwan Normal University, Taipei, Taiwan Department of Life Science, National Taiwan Normal University Taipei Taiwan

**Keywords:** Distribution, evolution, molecular, morphology, ND2 gene, systematics, taxonomy

## Abstract

Among the six species of *Cyrtodactylus* occurring in Sumatra, two species were described based on non-Sumatran type series, *C.consobrinus* and *C.quadrivirgatus*. The latter species was described originally from Thailand thus the wider distribution in Sumatra should be clarified taxonomically. *Cyrtodactylusquadrivirgatus* from Sumatra Barat was examined using both morphology and the Natrium Dehydrogenase Subunit 2 (ND2) gene to clarify its taxonomic status and phylogenetic placement. It was found that these specimens form a sister clade to all other species of the *sworderi* group from Peninsular Malaysia and the genetic distance ranges from 20–24.3%. This subset is herein described as a new species. The new species is readily distinguished from *C.quadrivirgatus* and other Sumatran species by a combination of characters: small size SVL 37.5–53.78 mm; longitudinal rows of dorsal tubercles 16–19; paravertebral tubercles 31–41; ventral scales 32–43; 24–49 enlarged precloacal and femoral scales; precloacal pores rarely present; no precloacal depression; two postcloacal tubercles on each side; 14–19 subdigital lamellae on forth toe; 9–15 supralabial scales; 9–12 infralabial scales; three or four internasal scales; and 3–6 gular scales that border the first pair of postmental scales. This work underscores the importance of clarifying widely distributed species for taxonomic validation.

## ﻿Introduction

*Cyrtodactylusquadrivirgatus* Taylor, 1962 was originally described from Khao Chong Forest Experiment Station, Trang Province, Thailand. It ranges from southern Thailand, Peninsular Malaysia and adjacent islands, Singapore to northern Sumatra (Grismer 2011) and Mentawai islands (Teynie et al. 2010), from sea level to 1400 m above sea level (Johnson et al. 2012).

Along Peninsular Malaysia, the populations of *C.quadrivirgatus* exhibit coloration differences among different localities. The south population has four dark dorsal stripes, the upland population has two dorsolateral stripes and medial blotches, and the other populations possess only blotches instead of stripes. Although there was obvious variation among populations, the ND2 p-distance showed that they were separated from each other by 3.3%–5.8% (Johnson et al. 2012).

Meanwhile, the population from Sumatra was not examined either morphologically or molecularly, leaving this population unknown in term of its taxonomic status and phylogenetic placement. We began surveying *Cyrtodactylus* Gray, 1827 in Sumatra Barat Province in 2020 and found them from lower elevations, approximately 8 m a.s.l. to 712 m a.s.l. Through careful examination, we wanted to establish whether *C.quadrivirgatus* from Sumatra Barat should be treated as a distinct species and into which lineage it fell.

## ﻿Material and methods

### ﻿Sampling and preservation

Field surveys were undertaken in the province of Sumatra Barat: Lembah Anai Nature Reserve (LANR) (0°29'24"S, 100°20'24"E), around Sarasah Gasang waterfall (SG) (0.31°S, 100.23°E), around Sungai Sirah village (SS) (0°24'8.8128"S, 100°8'37.6728"E), around Sarasah Uwak waterfall (0°54'28"S, 100°28'54"E) and in Bungus Selatan village (1°02'20"S, 100°24'50"E). Individuals were all collected during the night from 19.00–23.00 hours by hand. Anaesthetization and euthanization were done using benzocaine and fixation using 10% formalin. The specimens were stored in 70% alcohol and the livers were stored in 95% ethanol. All photographs were deposited at the Department of Biology, Universitas Negeri Padang, Indonesia (**UNP**). All specimens will be deposited at Museum Zoologicum Bogoriense, Bogor, Indonesia (**MZB**).

### ﻿Morphological analysis

Color notes were observed from digital images of living individuals prior to preservation. If in case the individual displayed stress coloration, we placed them in the cage mimicking the natural habitat and waited until the natural coloration appeared. The individuals under the stress condition showed black coloration along their dorsum, causing the disappearance of the black stripes and bands on the dorsum.

The following measurements were taken with a dial caliper to the nearest 0.5 mm following Hartmann et al. (2016) and Johnson et al. (2012):

**SVL** Snout-vent length, measured from the tip of snout to the vent;

**AX** Axial length, measured from the posterior margin of the forelimb at its insertion point on the body to the anterior margin of the hind limb at its insertion point on the body;

**TL** Tail length, measured from the vent to the tip of the tail, original or regenerated;

**AL** Arm length, measured insertion of antebrachium with body wall to claw of longest finger;

**LL** Leg length, measured insertion of femur with body wall to claw of longest toe;

**HL** Head length, measured from tip of snout to articulation of quadrate bone;

**HW** Head width, measured at level of ear openings;

**HH** Head height, measured at level of ear opening;

**SL** Snout length, measured from tip of snout to anterior margin of orbit;

**OEL** Orbit-ear length, measured from posterior margin of orbit to anterior margin of ear opening;

**OD** Orbit diameter, measured from anterior to posterior margin of orbit;

**EL** Ear length, measured from anterior to posterior margin of ear opening;

**ML** Mental length, maximum length of mental shield;

**IN** Internarial distance, measured between the nares across the rostrum;

**EN** Eye to nostril distance, measured between the anterior margin of the eyeball to the posterior margin of the external nares.

Meristic counts included:

**DTR** Dorsal tubercles, number of tubercle rows on dorsum at midbody, counted in one row between lateral folds;

**PVT** Paravertebral tubercles, number of tubercles counted in a longitudinal row between posterior insertion of fore limb and anterior insertion of hind limb;

**VS** Ventral scales, number of ventral scales at midbody, counted in one row between lateral folds;

**EPFS** Enlarged precloacal and femoral scales, number of enlarged precloaco-femoral scales, counted along lowest, pore-bearing row;

**PP** Precloacal pores, number of precloacal pores;

**PFP** Precloacal and femoral pores, number of precloaco-femoral pores;

**PCT** Postcloacal tubercles, number of postcloacal tubercles;

**LT4** Subdigital lamellae under 4^th^ toe, subdigital scales under 4^th^ toe, counted from first enlarged scale (true lamellae) on lower side of toe to scale proximal to apical scale;

**SLL** Left supralabial, labial scales of upper jaw, beginning with first enlarged scale bordering rostral shield, ending with last enlarged scale bordering labial angle for left side;

**SLR** Right supralabial, labial scales of upper jaw, beginning with first enlarged scale bordering rostral shield, ending with last enlarged scale bordering labial angle for right side;

**ILL** Left infralabial, labial scales of lower jaw, beginning with first scale bordering mental shield, ending with last enlarged scale bordering labial angle for left side;

**ILR** Right infralabial, labial scales of lower jaw, beginning with first scale bordering mental shield, ending with last enlarged scale bordering labial angle for right side;

**IN** Internasal scales, number of scales between rostronasals, bordering rostral shield;

**GUL** Gular scales, number of gular scales bordering pair of 1^st^ postmentals (excluding enlarged second 2^nd^ postmentals).

To make clear the counting of scales (supralabials and infralabials, precloaco-femoral scales) and detecting the presence of pores, we used a staining technique with methylene blue in 70% alcohol (Harvey et al. 2015). We determined male specimens by the enlarge hemipenial pockets and then confirmed the identification by making a small incision laterally at the base of the tail (Riyanto et al. 2021).

### ﻿Laboratory protocols

Total genomic DNA was extracted from the livers using the Qiagen DNeasy tissue kit (Valencia, CA, USA) following the standard protocol for animal tissue. The amplification of the Natrium Dehydrogenase Subunit 2 (ND2) gene and partial flanking tRNAs was done by using Polymerase Chain Reaction (PCR) under the following condition: 2 min at 95 °C followed by 33 cycles of 95 °C for 35 s, annealing at 54 °C for 35 s, extension at 72 °C for 35 s and a final extension step of 10 min at 72 °C. Amplifications were carried out in 25-µl volume vials consisting of 2.5 µl genomic DNA (concentration: approximately 100 ng), 0.4 µм each primer and 1× GoTaq Green Master Mix (Promega, Wisconsin, USA). The primers used in this study followed Macey et al. (1999a): L4437b (5’–AAGCAGTTGGGCCCATACC–3’) and L5002 (5’–AACCAAACCCAACTACGAAAAAT–3’). The PCR product was then sent to the sequencing service 1^st^ BASE (https://base-asia.com/) through Genetika Science Indonesia Limited Liability Company. The previous two primers were also used for sequencing.

### ﻿Phylogenetic reconstruction

Sequences were uploaded, assembled, and edited in Geneious Prime 2022.2.2 (http://www.geneious.com/). All sequences, ingroup and outgroup (Table 1), were aligned using CLUSTALW implemented in CIPRES Science Gateway. The fasta output of alignment was used for RAxML and uncorrected p-distance calculation. We reconstructed phylogenetic relationships using maximum likelihood analysis that was performed using RAxML HPC Black Box (1000 bootstrap replicates) implemented in CIPRES Science Gateway portal (Miller et al. 2010; accessed through https://www.phylo.org/). Nodal support with bootstrap value ≥ 70 was considered as significantly supported (Hillis and Bull 1993). The tree resulted from RAxML was visualised and edited in iTOL v. 6 (Letunic and Bork 2021; available at https://itol.embl.de/) and in Photoshop C6 64-bit. We also calculated uncorrected p-distances using MEGA 7 with delete option for the gaps (Kumar et al. 2016).

**Table 1.** Species of *Cyrtodactylus* used in the phylogenetic reconstruction including localities and GenBank accession numbers of the mitochondrial NADH dehydrogenase subunit 2 gene. PM= Peninsular Malaysia; Gn.= Gunung.

Species	Locality	Museum number	Accession number	Source
***agamensis* group**				
*C.metropolis*	Batu caves, Selangor, PM	LSUHC 11343	KU253579	Grismer et al. 2016
*C.payacola*	Bukit Panchor, Penang, PM	LSUHC 10070	JQ889190	Johnson et al. 2012
*C.majulah*	Nee Soon Swamp, Singapore	ZRC 26951	JX988529	Grismer et al. 2013
*C.pantiensis*	Gn. Panti, Johor, PM	LSUHC 8905	JQ889186	Johnson et al. 2012
*C.tiomanensis*	Pahang, PM	LSUHC 6251	JX440563	Wood et al. 2012
*C.rosichonariefi*	Bunguran, Great Natuna, Indonesia	MZB Lace 12132	KP256187	Riyanto et al. 2015
*C.psarops*	Indonesia	MZB 9687	MH248931	O’Connell et al. 2019
*C.* sp. 3	Indonesia	ENS 18140	MH248911	O’Connell et al. 2019
*C.* sp. 4	Indonesia	ENS 18591	MH248912	O’Connell et al. 2019
*C.* sp. 5	Indonesia	ENS 18659	MH248916	O’Connell et al. 2019
*C.* sp. 6	Indonesia	ENS 18719	MH248917	O’Connell et al. 2019
*C.semenanjungensis*	Gn. Panti, Johor, PM	LSUHC 8900	JQ889177	Johnson et al. 2012
*C.semicinctus*	Indonesia	ENS 14749	MH248925	O’Connell et al. 2019
C. cf. agamensis	Indonesia	ENS 19634	MH248908	O’Connell et al. 2019
***sworderi* group**				
*C.quadrivirgatus*	Bukit Larut, Perak, PM	LSUHC 8859	JQ889241	Johnson et al. 2012
*C.guakanthanensis*	Gua Kanthan, Perak, PM	LSUHC 11323	KU253577	Grismer et al. 2016
*C.tebuensis*	Gn. Tebu, Terengganu, PM	LSUHC 10902	JX988527	Wood et al. 2012
*C.sworderi*	Sungai Kawal, Peta, PM	LSUHC 7685	JQ889189	Johnson et al. 2012
*C.gunungsenyumensis*	Hutan Lipur Gn. Senyum, Pahang, PM	LSUHC 12201	KU253585	Grismer et al. 2016
*C.awalriyantoi* sp.nov.	Sungai Geringging, Padang Pariaman	UNP 153	OR122991	This study
		UNP 161	OR122987	
		UNP 162	OR122988	
		UNP 163	OR122989	
		UNP 164	OR122990	
***lateralis* group**				
*C.lateralis*	Indonesia	UTA 62916	KU893163	Harvey et al. 2016
*C.rubidus*	–	CES 131445	KM255203	Agarwal et al. 2014
*C.durio*	Malaysia	LSUHC 9725	KU893159	Harvey et al. 2016
***marmoratus* group**				
*C.marmoratus*	Indonesia	ENS 15932	KR921721	Harvey et al. 2015
*C.papuensis*	–	SAMA R62652	JQ820320	Oliver et al. 2012
*C.* sp. 1	Indonesia	ENS 15813	KR921697	Harvey et al. 2015
*C.* sp. 2	Indonesia	ENS 15784	KR921689	Harvey et al. 2015
***darmandvillei* group**				
*C.batucolus*	Pulau Besar, Melaka, PM	LSUHC 8934	JQ889179	Johnson et al. 2012
*C.petani*	Pasuruan, Jawa Timur, Indonesia	MZB Lace 11706	KU232620	Grismer et al. 2016
*C.kimberleyensis*	Siuna, Sulawesi Tengah, Pulau Sulawesi, Indonesia	WAM R164144	JX440544	Wood et al. 2012
*C.jellesmae*	Siuna, Sulawesi Tengah, Pulau Sulawesi, Indonesia	RMB 1672	GU550721	Siler et al. 2010
*C.sadleiri*	Christmas island, Australia	SAMA R34810	JQ820309	Oliver et al. 2012
*C.seribuatensis*	Pulau Nangka Kecil, Johor, PM	LSUHC 6349	JQ889187	Johnson et al. 2012
*C.darmandvillei*	Nusa Tenggara Barat, Indonesia	WAM R98393	JX440533	Wood et al. 2012
**Outgroup**				
*Hemidactylusfrenatus*	–	LLG 4871	GQ458049	Murthy et al. 2015
*Gekkogecko*	Thailand: Patong Beach, Kathu District, Phuket Island, Phuket Province	MVZ 215314	AF114249	Macey et al. 1999b

## ﻿Results

### ﻿Phylogenetic relationship of *Cyrtodactylus* from Sumatra Barat

**Figure 1.**
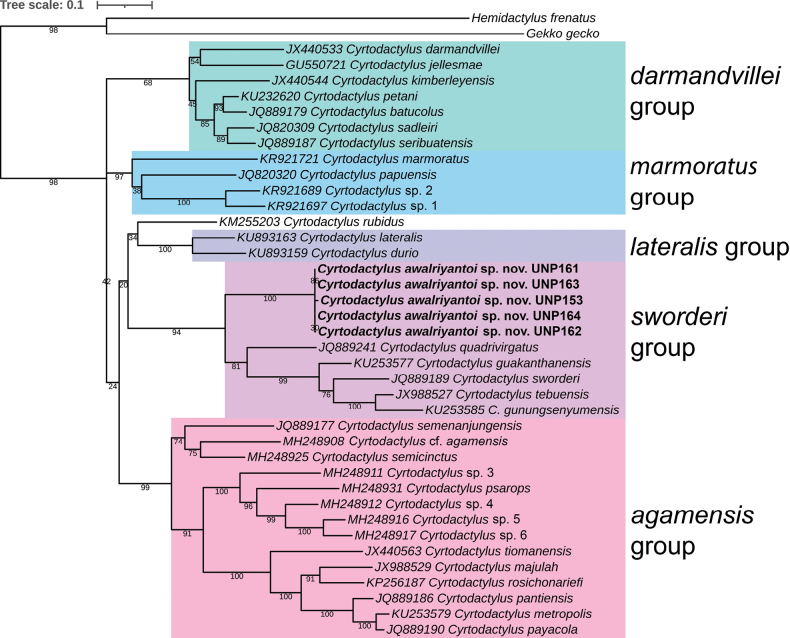
The maximum likelihood (ML) tree topology of the new species with other *Cyrtodactylus* inferred by ND2 gene sequences. The bold name within the *sworderi* group indicates new species that is being described. The numbers beneath the branches are bootstrap values.

We used 969–1005 bases of ND2 gene sequence from the new putative species to build a ML phylogenetic tree. Our ML tree (Fig. 1) showed that the new putative species is a sister clade of the *sworderi* group from Peninsular Malaysia (BS = 94) and it is a new member of *sworderi* group. The new putative species significantly formed a group (BS = 100). The uncorrected pairwise distance within this new putative species for ND2 gene is 0–0.5%. The distance from other species in the *sworderi* group ranges from 20% to 24.3% and from the *agamensis* group more than 30% (Table 2). Given these results, we further examined the morphological characters and found several distinctive characters. This subset is herein described as a new to science.

**Table 2.** Uncorrected p-distance (in %) of the ND2 gene calculated for the new species and *sworderi* and *agamensis* groups. For the accession numbers for each species, refer to Table 1.

No.	Species	1	2	3	4	5	6	7	8
1	*C.awalriyantoi* sp. nov.	0–0.5							
2	*C.quadrivirgatus*	20–20.6							
3	*C.guakanthanensis*	22.8–23.5	19.5						
4	*C.sworderi*	23.7–24.3	21.7	15.2					
5	*C.tebuensis*	20.9–21.5	20.2	13.4	17.1				
6	*C.gunungsenyumensis*	22.5–23.2	20.4	14.3	17.1	7.7			
7	*C.semenanjungensis*	32.3–33.1	26.5	31.4	32.1	30.4	28.3		
8	*C.semicinctus*	30.4–31.1	23.4	25.4	28.6	29.2	26.8	17.3	
9	*C.psarops*	33.6–34.3	27.4	28.3	35	31.3	26.8	26.1	22.7

### ﻿Taxonomy

#### ﻿ Cyrtodactylus awalriyantoi sp. nov.

Taxon classificationAnimaliaSquamataGekkonidae

E415127D-8B5F-576C-A05B-5F7BACB9AF72

https://zoobank.org/0D596952-07C2-439C-930C-D96C01C03F7C

Figs 2 , 3 , 4 , 5 , 6 , 7 , 8 , 9 Recommended English common name: Awal Riyanto’s Bent-toed Gecko Recommended Indonesia common name: Cicak Jari Lengkung Awal Riyanto

##### Type material.

**Figure 2.**
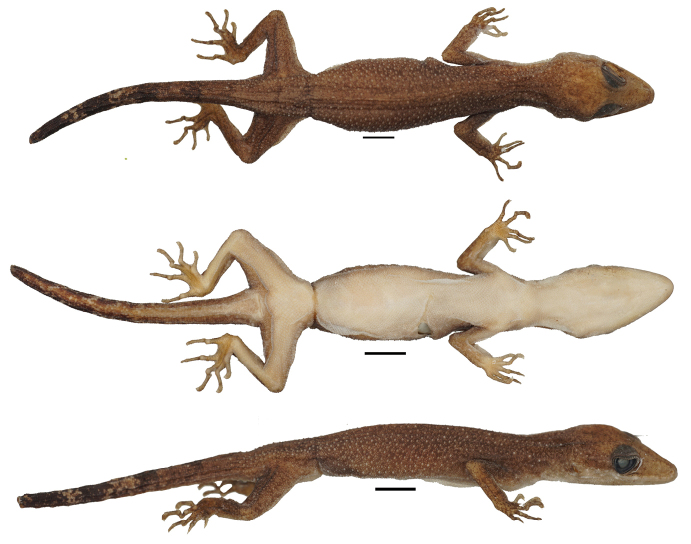
The holotype specimen (UNP070), adult male, of *Cyrtodactylusawalriyantoi* sp. nov. in preservation color. Scale bars: 10 mm.

***Holotype*.** Adult male, UNP070 (Fig. 2), collected from Sarasah Gasang waterfall (0°18'24.25"S, 100°13'45.15"E), Maninjau village, sub-district Tanjung Raya, regency of Agam by Y. Amardi, F. Lestari, and M. Kentino on 31 October 2020 at c. 08.30 pm. ***Paratypes*** (*N* = 17). Four individuals: three females (UNP103, 142, 143) and one male (UNP104), were collected from Lembah Anai Nature Reserve (0°29'24"S, 100°20'24"E), Singgalang village, Sepuluh Koto sub-district, Regency of Tanah Datar, province of Sumatra Barat by M. Rafi, K. Agusdi, F. Rozi, K. Agusdi, and F. A. D. Nugraha on 15 February 2022. Nine individuals: 5 females (UNP069, 075, 073, 067, 072) and 3 males (UNP066, 065, 071) collected from the same locality as holotype. Five individuals: 1 female (UNP153) and 4 males (UNP161, UNP162, UNP163, UNP164) collected from Sungai Sirah village (0°24'8.8128"S, 100°8'37.6728"E), sub-district Sungai Geringging, district Padang Pariaman on 7 May 2022 by M. Rafi and Y. Amardi.

##### Diagnosis.

*Cyrtodactylusawalriyantoi* sp. nov. is assigned to the *sworderi* group based on its phylogenetic position (Fig. 1) and the genetic distances to other congenerics (Table 2). This new species can be differentiated from all other *Cyrtodactylus* by having the following combination of characters: a small size, SVL 37.5–53.78 mm; axilla to groin distance 16.65–24.31 mm; head width 6.21–8.45 mm; longitudinal rows of dorsal tubercles 16–19; paravertebral tubercles 31–41; ventral scales 32–43; 24–49 enlarged precloacal and femoral scales; precloacal pores rarely present, maximum only two pores in one individual (only two individuals possessed pores); no precloacal groove or depression; postcloacal tubercles two on each side; 14–19 subdigital lamellae on fourth toe; 9–15 supralabial scales; 9–12 infralabial scales; 3–4 internasal scales; and 3–6 gular scales that bordered first pair of postmental scales.

##### Comparison.

This species is the smallest *Cyrtodactylus* species inhabiting Sumatra with the maximum SVL of adult individual of 53.78 mm. It can be distinguished from other *Cyrtodactylus* as follows:

1. *C.quadrivirgatus* by having following combination of characters: shorter maximum SVL (53.78 vs. 67 mm); shorter maximum length of axilla to groin (24.31 vs. 34 mm); shorter maximum length of tail (54.77 vs. 77 mm); shorter maximum length of arm (19.47 vs. 21 mm); shorter maximum length of leg (22.75 vs. 26 mm); shorter maximum length of head (15.42 vs. 18 mm); shorter maximum width of head (8.45 vs. 13 mm); shorter maximum length of snout to arm (21.43 vs. 32 mm); fewer DTR (16–19 vs. 24); maximum number of PVT is 41 (vs. 39); 2 precloacal pores (only in two specimens), mostly lack of pores in femoral and precloacal (vs. up to 12); fewer subdigital lamellae on 4 ^th^ toe (14–19 vs. 18–23); maximum number of supralabial (15 vs. 11); maximum number of IN scales is 4 (vs. 3); and lack of black stripe between eyes and naris (vs. present).
2. *C.psarops* by lower number of DTR (16–19 vs. 28–38); greater number of PVT (31–41 vs. 23–26); minimum number of VS of 32 (vs. 38); number of PCT (2 on each side vs. 1 on each side); fewer subdigital lamellae on 4 ^th^ toe (14–19 vs. 18–22); lacking precloacal groove/depression (vs. present); pores rarely present and maximum of 2 pores (vs. 28–32); lacking U-shaped band on occiput/nuchal; and having extended lateral stripe (vs. lacking).
3. *C.semicinctus* by lower number of DTR (16–19 vs. 29–35); maximum number of PVT of 41 (vs. 35); maximum number of VS of 40 (vs. 44); maximum number of PCT of 2 on each side (vs. 3 on each side); fewer subdigital lamellae on 4 ^th^ toe (14–19 vs. 19–22); lacking precloacal depression (vs. present); pores rarely present and maximum of 2 pores (vs. 36–38); brachium tuberculated (vs. not tuberculated); and having extended lateral stripe (vs. lacking).
4. *C.lateralis* by having more PVT (31–41 vs. 21–28); fewer VS (32–43 vs. 51–66); 0–2 precloacal pores and rarely present (vs. 9–15); fewer subdigital lamellae on 4 ^th^ toe (14–19 vs. 18–24); lacking spinose tubercles in caudal region, conical tubercles in the ventrolateral fold; and lacking a prehensile tail.
5. *C.consobrinus* by having fewer VS (32–43 vs. 58–65); fewer precloacal pores (0–2 vs. 9–10); fewer subdigital lamellae on 4 ^th^ toe (14–19 vs. 23–28); lacking narrow light line like network on the head; having extended lateral stripe (vs. lacking); and lacking white crossbands on the dorsum.
6. *C.agamensis* by lower number of DTR (16–19 vs. 50–67); greater maximum number of PVT (41 vs. 37); pores rarely present and maximum of two pores (vs. 9–10); lower number of subdigital lamellae under the 4 ^th^ toe (14–19 vs. 21–26); 15 supralabial scales (vs. 13); and having four dorsal stripes (vs. absence).

*C.awalriyantoi* sp. nov. has unique morphological combination and can be separated from other congeners within the *sworderi* group as follows:

1. *C.gunungsenyumensis* by shorter SVL (37.5–53.78 mm vs 65.1–74.7 mm); fewer subdigitall lamellae on fourth toe (14–19 vs 20–23); and more enlarged precoloaco femoral scales (24–49 vs 31–39).
2. *C.tebuensis* by shorter SVL (37.5–53.78 mm vs 73.1–84.1 mm); fewer ventral scales (32–43 vs 43–51); fewer subdigital lamellae on fourth toe (14–19 vs 17–21); more infralabial scales (9–12 vs 8–10); and more enlarged precoloaco femoral scales (24–49 vs 31–38).
3. *C.sworderi* by shorter SVL (37.5–53.78 mm vs 69–80 mm); and fewer ventral scales (32–43 vs 42–49)
4. *C.guakanthanensis* by shorter SVL (37.5–53.78 mm vs 82.2 mm); more supralabial (9–15 vs 9–10); more infralabials (9–12 vs 7–8); fewer subdigital lamellae on fourth toe (14–19 vs 19–21); and more enlarged precloaco-femoral scales (24–49 vs 36–41).

##### Description (and variation).

**Figure 3.**
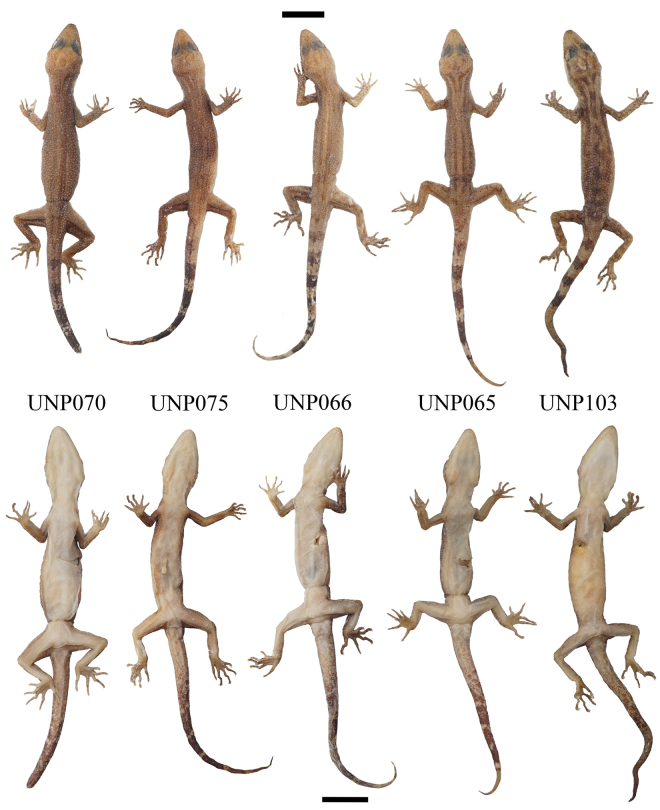
Representative type series of *C.awalriyantoi* sp. nov. after preservation. UNP070 (male); UNP075 (female); UNP066 (male); UNP065 (male); UNP103 (female). Scale bars: 10 mm; upper for dorsal view and lower for ventral view.

**Figure 4.**
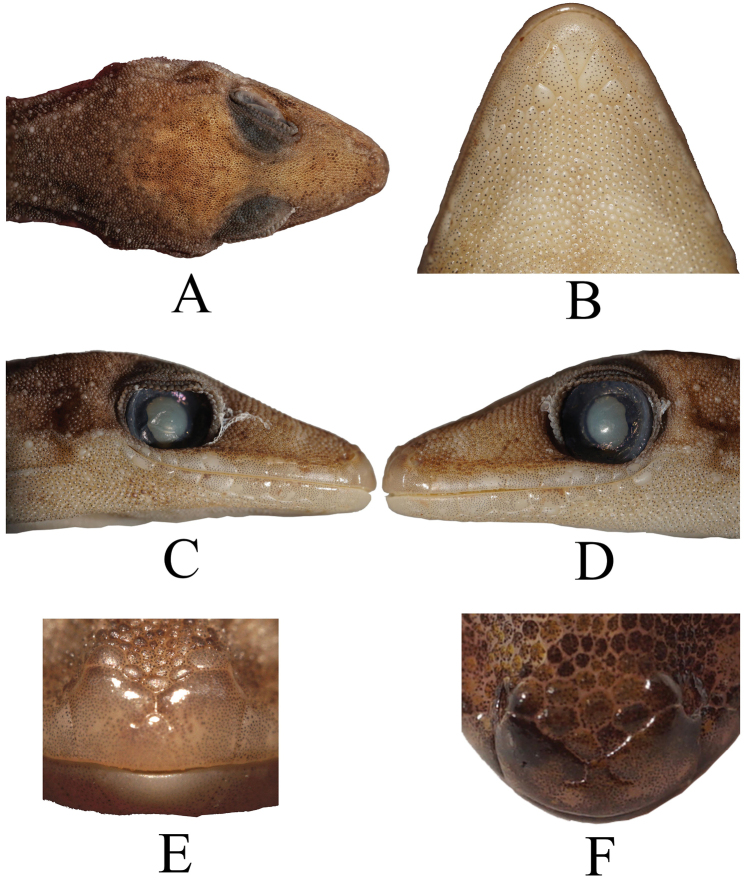
The head of holotype specimen (UNP070) of *Cyrtodactylusawalriyantoi* sp. nov. **A** dorsal view **B** ventral view **C**, **D** lateral views of right and left, respectively. Images not to scale.

Small-sized *Cyrtodactylus* with SVL of 37.5–53.78 mm; the length of the tail is 31.4–54.77 mm including the original or regenerated tip; the axial body length is 16.65–24.31 mm (Fig. 3). The head is triangular in dorsal view with moderate length (HL/SVL= 0.25–0.31), wide (HW/HL 0.52–0.63), and slightly flattened (HH/HL= 0.29–0.45), distinguishable from neck; medium length of snout (SL/HL 0.33–0.45) and rounded; snout longer than eye diameter (SL/OD 1.38–2.03); eyes large (OD/HL 0.18–0.27); ear openings oval and small (EL/HL 0.02–0.1); eye to ear distance greater than diameter of eye (OEL/OD 0.89–1.54); postorbital and around ear region consists of enlarged tubercles; scales on post nasal to preorbital and post-rostral to frontal region slightly larger in size than scales on the parietal part and occiput; region of parietal containing small scales intermixed with weak, scattered, rounded tubercles while occiput region contained slightly enlarged tubercles (Fig. 4).

The nares are oval, bordered by rostral anteriorly, by supranasals and internasals dorsally, by 1^st^ supralabial ventrally. Supranasal scales larger than post-nasal scales. The supranasal scales as large as intersupranasals and separated from each another by three or four intersupranasal scales (Fig. 4F).

**Figure 5.**
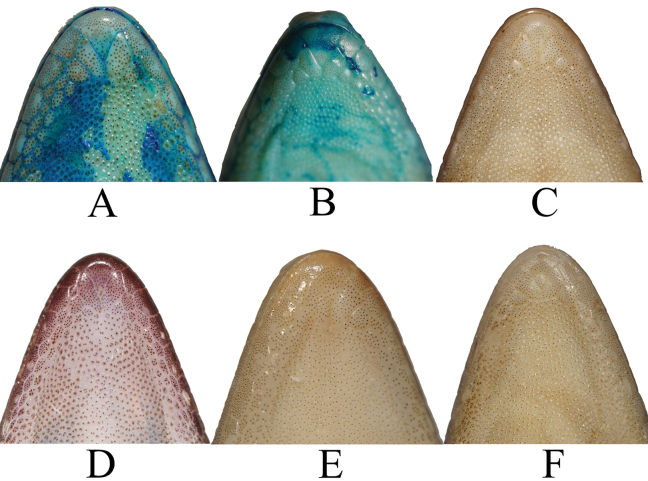
Ventral view of head showing second postmental variations in which they attach to the supralabials and variation in number of smaller scales separating them **A** UNP067 **B** UNP073 **C** UNP070 **D** UNP142 **E** UNP069 **F** UNP072. Images not to scale.

The triangular mental is bordered laterally by first infralabial and posteriorly by right and left first postmental. First postmentals medially connected each other for ~ 30% of their length. Second postmentals in contact with 1^st^ and 2^nd^ infralabials (*N* = 2) (Fig. 5A), separated from infralabials by relatively smaller scales (*N* = 1) (Fig. 5B), by relatively similar-sized scales (*N* = 5) (Fig. 5C), and by relatively larger scales (*N* = 1) (Fig. 5D). Right scale contacts with 1^st^ and 2^nd^ infralabials but the left only with 2^nd^ infralabial (*N* = 2) (Fig. 5E), or the right contacted with 2^nd^ infralabial and the left with small part of 1^st^ infralabial and large part of 2^nd^ infralabial (Fig. 5F). Right and left second postmentals are bordered by 3–6 relatively smaller scales (Fig. 5).

**Figure 6.**
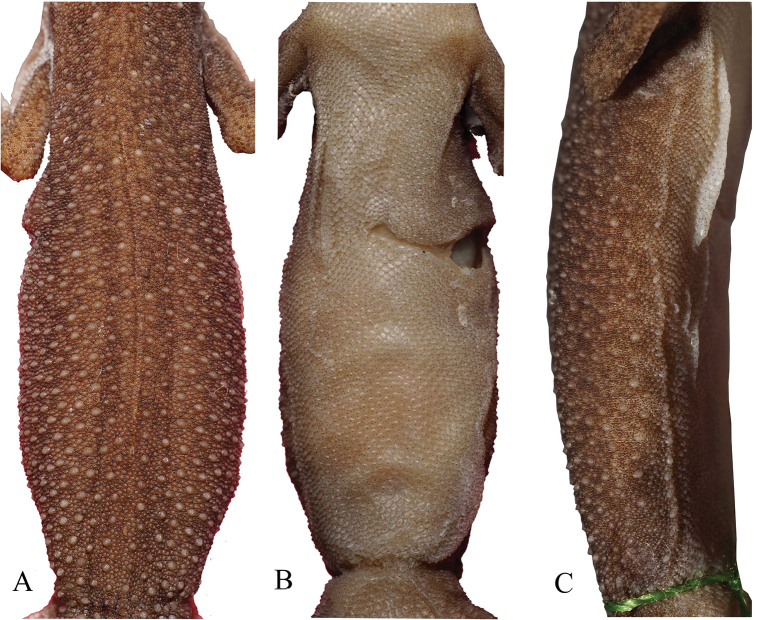
Trunk in **A** dorsal **B** ventral **C** ventrolateral views of holotype specimen (UNP070).

Body moderate in length (AX/SVL 0.38–0.53); defined ventrolateral fold with tubercles smaller than dorsal tubercles; dorsum with small scales interspersed with large conical or pyramidal, tubercles most dense on flanks; tubercles extending from occipital region to the base of tail, tubercles on tail largest; 16–19 tubercles between lateral fold in middle of body; 31–41 tubercles of paravertebral from posterior insertion of arm to body to anterior of femur insertion to body; 32–43 ventral scales larger than dorsal scales; ventral scales in middle part slightly larger than those near the ventrolateral folds; from middle of body, scales are smaller anteriorly to the head, ventrum, and posteriorly until groin region (Fig. 6).

**Figure 7.**
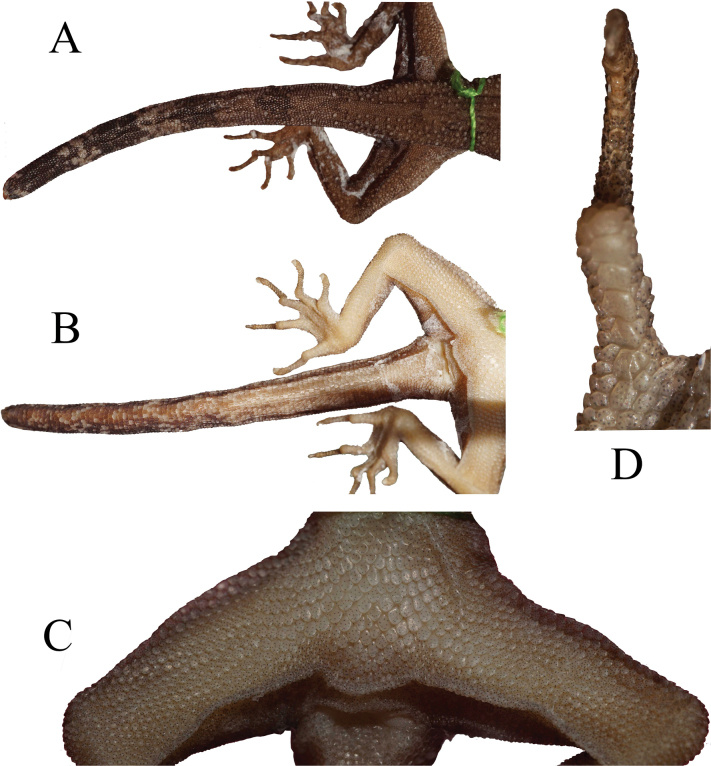
Tail in dorsal **A** and ventral **B** views, precloacal and femoral view (**C**) and ventral of 4^th^ toe (**D**). All images from holotype specimen (UNP070). Images not to scale.

Forelimbs medium length (AL/SVL 0.33–0.4); granular scales on upper arm larger than those on dorsum of body (~ 2–3 ×larger); without tubercles; lower arm with smaller scales than upper arm scales, intermixed with weak tubercles slightly larger than weak tubercles on parietal parts; hindlimbs also moderate in size (LL/SVL 0.40–0.52); more robust than forelimbs; covered dorsally by granular scales intermixed with large, rounded tubercles; ventral scales of thigh larger than dorsals; 14–19 subdigital lamellae on 4^th^ toe. Continuous enlarged precloacal and femoral scales present (*N* = 24–49); no specimen has precloacal groove/ depression; enlarged post-precloacal scales present; two post-cloaca tubercles on left and right base of tail, mostly connected to each another (Fig. 7).

Tail length ~ 1.1 × of SVL, circular in cross-section but tapering at the end portion; tubercles on base of tail dorsally similar in size to those on body dorsum; 4–11 black dorsoventral stripes separated by white stripes; black stripes on venter more faded than on dorsal; part; no median, transversely enlarged, series of scales on the subcaudal; subcaudal cycloidal scales relatively larger than dorsal (Fig. 7).

##### Coloration in life.

**Figure 8.**
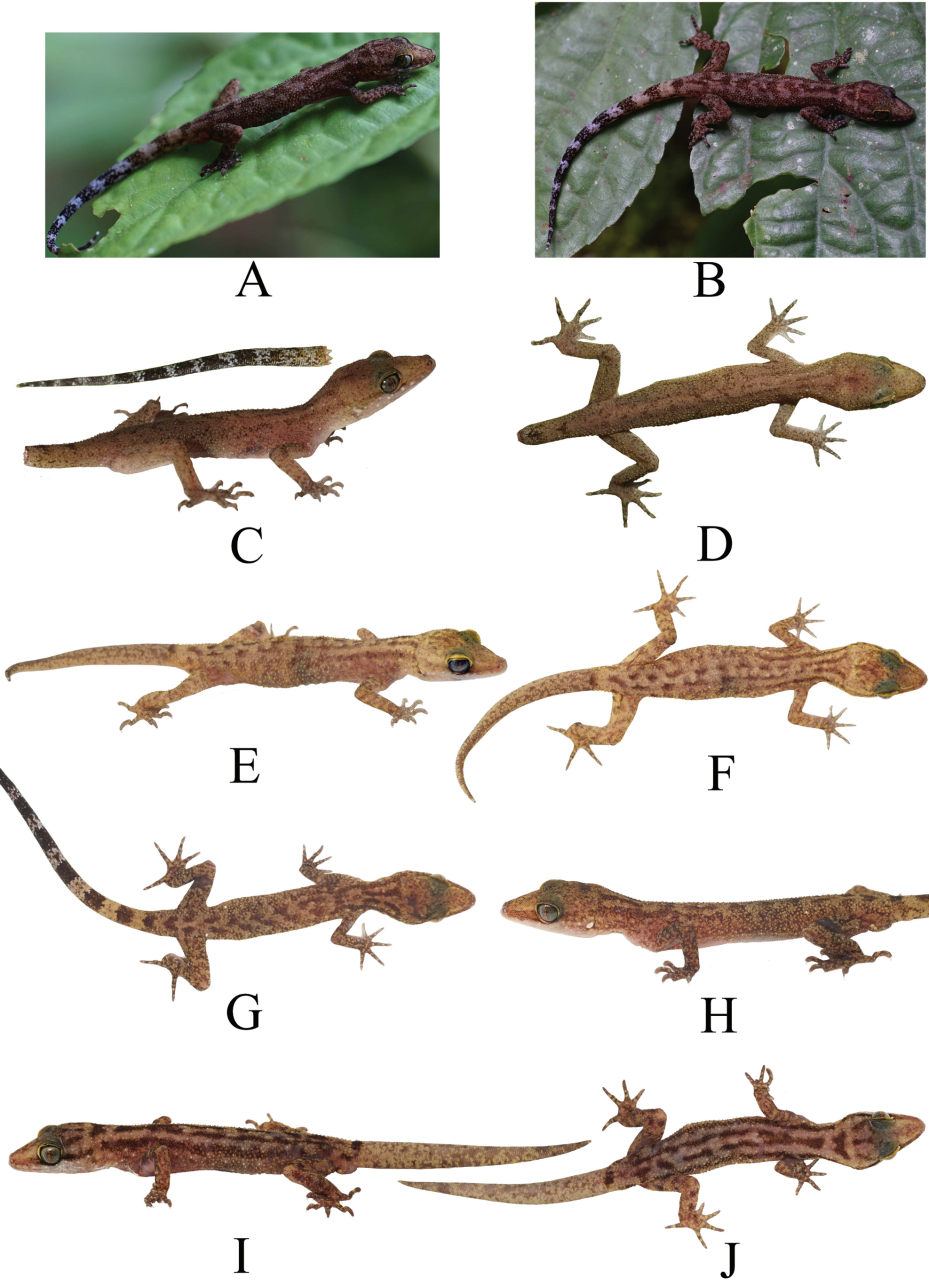
Coloration in life of *C.awalriyantoi* sp. nov. **A, B** UNP142 **C, D** UNP143 **E, F** UNP153 **G, H** 162 **I, J** unvouchered specimen. Images not to scale.

Ground color of body dorsum dark grey to brown; top of head blackish with irregular broken spots scattered on parietal region to nostril; on occipital regions three short black lines extending longitudinally: one in the middle, two begin behind each side of eyes almost parallel to the supraorbital regions; those three short black bands stop at approximately parallel to ears, after which there is a transverse white line extending from each pre-ear region; after the white line, there are two black lines at the nape of the neck that extend backwards, then some meet at an angle and some remain separate, as if these two lines continue the black line originating from the back of the eye parallel to the supraorbital area; after the meeting, there are two lines that separate to the back of the tail, and some are still united to the tail so that it tends to look like a black transverse band; in individuals with the two midlines converging, the confluence of the two lines begins just before the anterior part of the upper arm; there are eight or nine rows of black transverse bands that are counted from the beginning of the union of the two lines to the base of the tail; on the dorsolateral, there are two black lines that extend from behind the eyes to the base of the tail; unpatterned black blotches or obscure irregular black banding on limbs; black and white bands on tails; the width of the black line increases towards the posterior; and the white is opposite; in some individuals, the above-mentioned black stripes are not clear and not strong along the dorsal and dorsolateral body. Ventral surface of head, trunk, and limbs are white, pale grey to cream; ventral surface of tail cream in the first third at the anterior, then the rest to the posterior tends to black with narrow white rings (Fig. 8).

##### Coloration in preservative.

Ground color of dorsal trunk, limbs, and tail brown to dark; parietal part to the tip of snout paler than any other parts of dorsum; the individuals with unclear or weak black lines on middle dorsum and dorsolateral tend to be dark from the nape to the base of the tails; black lines on nape and trunk still visible; tail with black and white bands; ventral head, trunk, limbs whitish to dark brown. Fresh specimens darker than the others both in ventral or dorsal parts of the body (Fig. 3).

##### Habitat.

**Figure 9.**
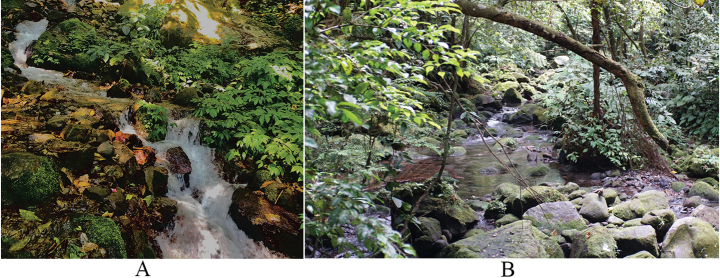
Habitat type of *Cyrtodactylusawalriyantoi* sp. nov. in Sarasah Gasang Waterfall (**A**) and Lembah Anai Nature Reserve (**B**).

**Figure 10.**
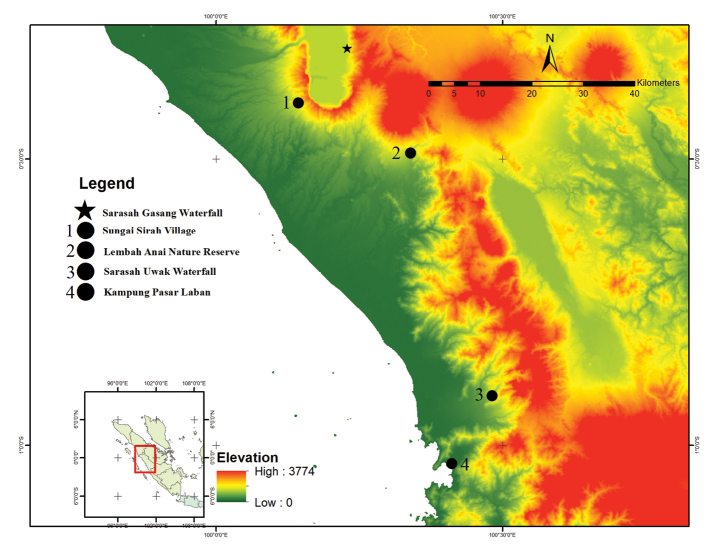
Type locality and distribution of *C.awalriyantoi* sp. nov. in Sumatra Barat Province.

We collected the type series in the primary forest of LANR, SG, and SS with elevation ~ 380–767 m a.s.l. and we encountered non-vouchered individuals from ~ 7 m a.s.l. At SG, this species was found on leaves measuring ~ 7–10 cm width and on twigs, ~ 1 m above the ground, 1–3 m from the edge of the rocky stream. The stream that empties into the waterfall has a breadth of ~ 2 m with a heavy flow. Fewer specimens were found closer to the waterfall. At LANR and SS, this species occupied the same microhabitat as the SG population, but the stream at this location is wider (~ 5–7 m width; Fig. 9). We also encountered this species (an unvouchered individual) at a lower elevation of 7 m a.s.l. in Bungus Selatan village. At this location, the gecko was perching on a bush leaf just beside the paddy field at ~ 70 cm above the ground. Another unvouchered individual was in the Sarasah Uwak waterfall area but far from the waterfall, perching on bushes at ~ 60 cm above the ground (Fig. 10).

##### Distribution.

Currently, this new species is found only in Sumatra.

##### Etymology.

The specific epithet *awalriyantoi* is in reference to the Indonesian herpetologist, Awal Riyanto. He has dedicated much of his time researching Indonesian *Cyrtodactylus* from Indonesia, as well as patiently and continuously supervising many younger amphibian and reptile taxonomists from both academic institutions and independent positions. Moreover, his contribution to the study of amphibians and other reptiles is significant for Indonesian herpetological knowledge and conservation.

## ﻿Discussion

Previously, the *sworderi* group of *Cyrtodactylus* contained five species of which four are endemic to Peninsular Malaysia: living in lowland swampy habitats (*C.sworderi*; Taylor 1962), upland habitats (*C.tebuensis*; Grismer et al. 2013), and in karstic habitats (*C.guakanthanensis* and *C.gunungsenyumensis*; Grismer et al. 2014, 2016). The fifth species, *C.quadrivirgatus*, is a habitat generalist that is widely distributed from Thailand to Sumatra (Grismer 2011). Our study revealed that *C.quadrivirgatus* from Sumatra Barat differs from the Peninsular Malaysian population based on molecular and morphological evidence. With this addition, Sumatra currently supports six species of *Cyrtodactylus* in total, but the number of species endemic to this mainland is five: *C.agamensis*, *C.lateralis*, *C.psarops*, *C.semicinctus* and *C.awalriyantoi*.

Widely distributed species in *Cyrtodactylus* are most likely questionable, for example, two potentially new species have been detected within the *C.marmoratus* complex from southern Sumatra (O’Connell et al. 2019). This study also showed that widely distributed species like *C.quadrivirgatus* need confirmation. At the current state of knowledge, only *C.consobrinus* has a wide distribution, originally described from Sarawak (Borneo) and reported from Sumatra (Teynie et al. 2010). Like the new species *C.awalriyantoi*, its presence in Sumatra most likely warrants taxonomic validation.

## Supplementary Material

XML Treatment for Cyrtodactylus awalriyantoi

## ﻿Additional information

### Author ORCIDs

Yuni Ahda https://orcid.org/0000-0003-0545-2965

Fitra Arya Dwi Nugraha https://orcid.org/0000-0002-0048-8515

Djong Hon Tjong https://orcid.org/0000-0002-4743-9964

Muhammad Alif Fauzi https://orcid.org/0000-0003-0975-9681

Si-Min Lin https://orcid.org/0000-0001-7080-706X

### Data availability

All of the data that support the findings of this study are available in the main text or Supplementary Information.

## Supplementary materials

Supplementary material 1 Morphometric and meristic data Data type Morphology (.xlsx file) Explanation note This file contain the measurement on morphometric characters and meristic, examined on the type series specimen. https://binary.pensoft.net/file/872858This dataset is made available under the Open Database License (http://opendatacommons.org/licenses/odbl/1.0/). The Open Database License (ODbL) is a license agreement intended to allow users to freely share, modify, and use this Dataset while maintaining this same freedom for others, provided that the original source and author(s) are credited.Yuni Ahda, Fitra Arya Dwi Nugraha, Djong Hon Tjong, Nia Kurniawan, Yunico Amardi, Muhammad Alif Fauzi, Si-Min Lin

Supplementary material 2 Comparison with *C.quadrivirgatus* Data type Morphology (.xlsx file) Explanation note This file contains the comparison between *C.awalriyantoi* sp. nov. with *C.quadrivirgatus* from type series (Taylor 1962) and from Peninsular Malaysia population (Johnson et al. 2012). https://binary.pensoft.net/file/872859This dataset is made available under the Open Database License (http://opendatacommons.org/licenses/odbl/1.0/). The Open Database License (ODbL) is a license agreement intended to allow users to freely share, modify, and use this Dataset while maintaining this same freedom for others, provided that the original source and author(s) are credited.Yuni Ahda, Fitra Arya Dwi Nugraha, Djong Hon Tjong, Nia Kurniawan, Yunico Amardi, Muhammad Alif Fauzi, Si-Min Lin
